# Single-cell transcriptomics reveals metabolic remodeling and functional specialization in the immune microenvironment of bone tumors

**DOI:** 10.1186/s12967-025-06346-0

**Published:** 2025-05-16

**Authors:** Jun Chen, Na Cui, Shao-Hui He, Chun-Yan Xia, Wei-Qing Li

**Affiliations:** 1 Department of Pathology, Changzheng Hospital, Naval Medical University, Shanghai, 200003 China; 2 Department of Orthopaedic Oncology, No.905 Hospital of People’s Liberation Army Navy, Changzheng Hospital, Naval Medical University, Shanghai, 200003 China

**Keywords:** ScRNA-seq, Bone tumors, Immune microenvironment, Metabolic remodeling

## Abstract

**Objective:**

To investigate the metabolic remodeling and functional specialization of immune cells within the tumor microenvironment (TME) of bone tumors, including Ewing’s sarcoma, osteosarcoma, and giant cell tumor of bone, through high-resolution single-cell RNA sequencing (scRNA-seq) analysis.

**Methods:**

Immune cells were isolated from 13 bone tumor samples and profiled via scRNA-seq to delineate cellular compositions, metabolic adaptations, and intercellular communication networks. Differential gene expression analysis, metabolic pathway enrichment, and pseudotime trajectory inference were employed to characterize functional states and differentiation processes of immune cell subsets.

**Results:**

We identified 12 major immune cell clusters with distinct functional and metabolic characteristics. Naïve T cells exhibited amino acid metabolism-dependent activation potential, whereas NK cells relied on lipid metabolism and the TCA cycle for cytotoxic activity. Macrophage subsets demonstrated functional divergence: C06 macrophages adopted lipid metabolism to facilitate immunosuppression and tissue repair, while C04 macrophages displayed pro-inflammatory characteristics associated with complement activation. Intercellular signaling analysis revealed FN1 as a central regulator of immune coordination, governing cell adhesion, migration, and homeostasis within the TME.

**Conclusion:**

This study provides novel insights into the metabolic and functional plasticity of immune cells in bone tumor TMEs, underscoring the critical role of metabolic remodeling in immune regulation. Our findings highlight potential therapeutic targets for modulating immune cell function and offering new avenues to improve treatment outcomes for patients with bone tumors.

**Supplementary Information:**

The online version contains supplementary material available at 10.1186/s12967-025-06346-0.

## Introduction

Bone tumors represent a highly heterogeneous group of malignancies, posing significant challenges to the medical community, impacting millions of patients worldwide, and profoundly affecting survivors’ quality of life [1]. Primary malignant bone tumors include Ewing’s sarcoma (ES) and osteosarcoma (OS), while giant cell tumor of bone (GCTB) is an intermediate-grade neoplasm with locally aggressive behavior [2]. However, early-stage primary bone tumors are often asymptomatic, leading to delayed medical consultation, typically prompted by pathological fractures or severe pain [3]. Unfortunately, the highly invasive nature of malignant bone tumors enables rapid progression and metastasis, particularly to the lungs, posing a major challenge for early diagnosis and effective intervention.

Despite substantial research efforts to identify molecular targets for treatment, no therapeutic strategies currently appear poised to transform the clinical management of primary bone malignancies [4]. Immunotherapy has emerged as a promising alternative, gaining increasing attention in oncology [5, 6]. Although the immune system possesses the intrinsic capacity to recognize and eliminate tumor cells, immune evasion remains a fundamental hallmark of cancer [7]. Thus, a comprehensive investigation of the tumor microenvironment (TME) is urgently needed to advance our understanding of systemic immunotherapy in primary bone tumors.

In this study, we employed single-cell RNA sequencing (scRNA-seq) to systematically delineate the immune landscape of bone tumors at single-cell resolution. By analyzing immune cells from tumor samples of ES, OS, and GCTB patients, we characterized cellular heterogeneity, functional specialization, and metabolic adaptations within the TME. Specifically, our analysis identified distinct immune cell populations, including macrophage subsets exhibiting either pro-inflammatory or tissue-remodeling phenotypes, as well as functionally diverse T cell subpopulations, revealing the dynamic interplay between immune activation and suppression. These findings provide new insights into immune regulatory mechanisms in bone tumors and highlight potential therapeutic targets for modulating the TME.

## Material and methods

### Data and code availability

The raw single-cell RNA sequencing (scRNA-seq) datasets analyzed in this study have been deposited in the Gene Expression Omnibus (GEO) under accession numbers GSE168664, GSE212341, GSE210750, and GSE198896. Detailed clinicopathological information for patients with ES, OS, and GCTB is available in the Supplementary Materials (ST.12). The R scripts used for primary data processing and analysis can be accessed upon reasonable request to the corresponding authors.

### Single-cell RNA-seq data processing

All analyses were conducted in R (version 4.0.3) using the Seurat package (version 3.1.1). During quality control, cells with fewer than 200 detected genes or a mitochondrial gene content exceeding 10% were excluded. Doublets or multiplets were identified and removed using the DoubletFinder function when multiple population-specific marker genes exhibited aberrantly high expression within a single cell. Additionally, cells with UMI counts exceeding 40,000 or gene counts above 5000 were excluded to mitigate potential doublet contamination. The remaining UMI counts were normalized using the NormalizeData function with the ‘logNormalize’ method and a scaling factor of 10,000.

### Dimensionality reduction and unsupervised clustering

Highly variable genes were identified using the FindVariableGenes function under default parameters and subsequently used for linear dimensionality reduction. Principal Component Analysis (PCA) was performed on the top 2000 highly variable genes using the RunPCA function. The optimal number of principal components was determined based on ElbowPlot inspection. For visualization, uniform manifold approximation and projection (UMAP) was applied using the RunUMAP function with a perplexity value of 30. Unsupervised clustering of single cells was conducted using the FindClusters function at a resolution of 0.6, leveraging the same principal components used for UMAP visualization.

### Identification of differentially expressed genes (DEGs)

Differentially expressed genes (DEGs) across clusters were identified using the FindAllMarkers or FindMarkers function in Seurat. Adjusted P-values were computed using the Bonferroni correction, and genes with adjusted P-values exceeding 0.05 were excluded. For pairwise differential expression analysis between immune cell subclusters, the Wilcoxon rank-sum test was employed.

### Metabolic pathway analysis

Metabolic pathway activity was inferred using the scMetabolism R package, which integrates single-cell expression data with KEGG-defined metabolic pathways. Pathway scores were calculated for each cell, and metabolic differences among subpopulations were visualized using heatmaps and violin plots. This analysis provided insights into the metabolic specialization and plasticity of immune cells within the bone tumor microenvironment.

### Pseudotime transcriptional trajectory analysis

Cellular differentiation trajectories were inferred using the Monocle2 package. The top 400 signature genes identified by the Differential GeneTest function were selected as input for pseudotime analysis. RNA expression counts from selected subclusters were utilized to reconstruct the developmental trajectories of monocytes, macrophages, osteoclasts, T and NK cells. Lineage differentiation was determined following dimensionality reduction and cell ordering, while generalized additive models (GAMs) were employed to visualize expression trends.

### Cell–cell interaction network analysis

Cell–cell communication was analyzed using the CellChat package to infer intercellular signaling interactions. The netAnalysis_signalingRole_scatter function was applied to visualize incoming and outgoing interaction strengths, whereas the netAnalysis_contribution function identified significant ligand-receptor pairs. Highly enriched signaling pathways were displayed using the netAnalysis_signalingRole_heatmap and netVisual_aggregate functions. Specific signaling interactions, such as FN1-mediated signaling between source and target cell types, were visualized using the netVisual_bubble function.

### Statistical analysis

All statistical analyses were performed using R software (version 4.0.3) and GraphPad Prism (version 8). Data were expressed as mean ± standard error of the mean (SEM). For comparisons between two groups, either an unpaired Student’s t-test or Wilcoxon rank-sum test was conducted, depending on data distribution. For comparisons across more than three groups, one-way analysis of variance (ANOVA) was applied. Statistical significance was defined as P-values < 0.05 (two-tailed).

## Results

### Immune landscape of the bone tumor microenvironment

**Fig. 1:**
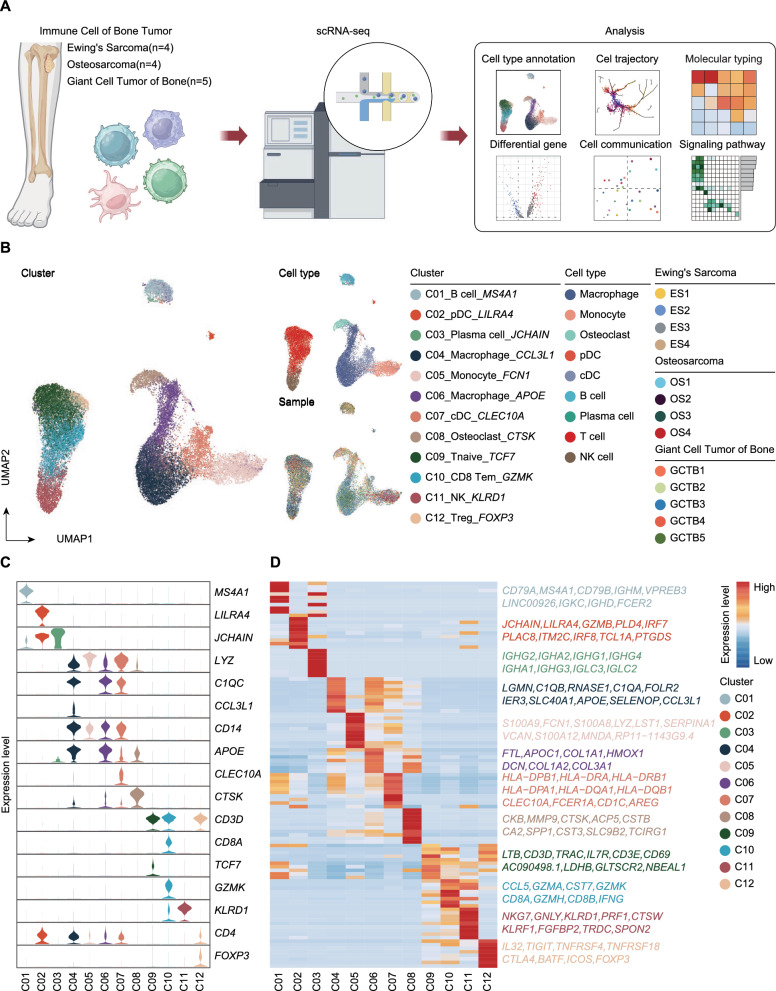
Workflow and single-cell RNA sequencing analysis reveal the immune microenvironment of bone tumors. **A** Schematic representation of the single-cell RNA sequencing workflow. **B** UMAP plot illustrating the clustering of immune cells from 13 tumor samples. The figure uses color coding to represent immune cell clusters, cell types, and sample origin. **C** Violin plots showing the expression of marker genes used to identify cell types and characteristic genes for each immune cell cluster. **D** Heatmap displaying the top 10 differentially expressed genes (DEGs) for each immune cell cluster

Through scRNA-seq analysis of 13 bone tumor samples from patients (4 ES, 4 OS, and 5 GCTB) (Fig. 1A), we identified 12 major immune cell clusters using UMAP-based clustering (Fig. 1B). These clusters included C05_Monocyte_*FCN1* (high CD14 expression), two macrophage subsets (C04_Macrophage_*CCL3L1* and C06_Macrophage_*APOE*), plasmacytoid dendritic cells (C02_pDC_*LILRA4*), classical dendritic cells (C07_cDC_*CLEC10A*), osteoclasts (C08_Osteoclast_*CTSK*), and B cells (C01_B cell_*MS4A1*). T cells were further subdivided into three functionally distinct subpopulations: naïve T cells (C09_Tnaïve_*TCF7*), memory T cells (C10_Tem_*GZMK*), and regulatory T cells (C12_Treg_*FOXP3*). Natural killer (C11_NK_*KLRD1*) cells exhibited high *KLRD1* expression (Fig. 1C).

Distinct functional characteristics were observed in macrophage and T cell subsets based on their top-enriched genes. C04 macrophages, characterized by high *CCL3L1* expression*,* exhibited a pro-inflammatory phenotype, with additional enrichment of genes such as *C1QB, FOLR2*, and *LGMN*, indicating roles in complement activation, antigen processing, and immune recruitment [8, 9]. In contrast, C06 macrophages, defined by high *APOE* expression, were associated with lipid metabolism and extracellular matrix remodeling, as indicated by the co-expression of *COL1A1*, *COL3A1*, and *HMOX1* [10]. These findings suggest functional specialization, with C04_Macrophage_*CCL3L1* promoting inflammation and immune activation, whereas C06_Macrophage_*APOE* contributing to stromal remodeling and metabolic regulation.

T cell subsets also displayed considerable heterogeneity and specialization within the tumor microenvironment. Naïve T cells (C09) marked by high expression of *TCF7*, *IL7R*, and *CD69*, played essential roles in early antigen recognition and differentiation [11]. Memory T cells (C10) exhibited high expression of *GZMK*, *CCL5*, and *IFNG*, reflecting their cytotoxic potential and effector memory functions [12]. In contrast, regulatory T cells (Tregs) (C12), characterized by high *FOXP3*, *CTLA4*, and *TIGIT* expression, played immunosuppressive roles in maintaining immune homeostasis and facilitating immune evasion [13] (Fig. 1D).

Our findings provide a comprehensive overview of immune cell diversity in bone tumors, revealing the functional specialization of macrophage and T cell subsets and their roles in shaping the tumor microenvironment.

### Immunological heterogeneity and subtype-specific immune dynamics in bone tumors

**Fig. 2:**
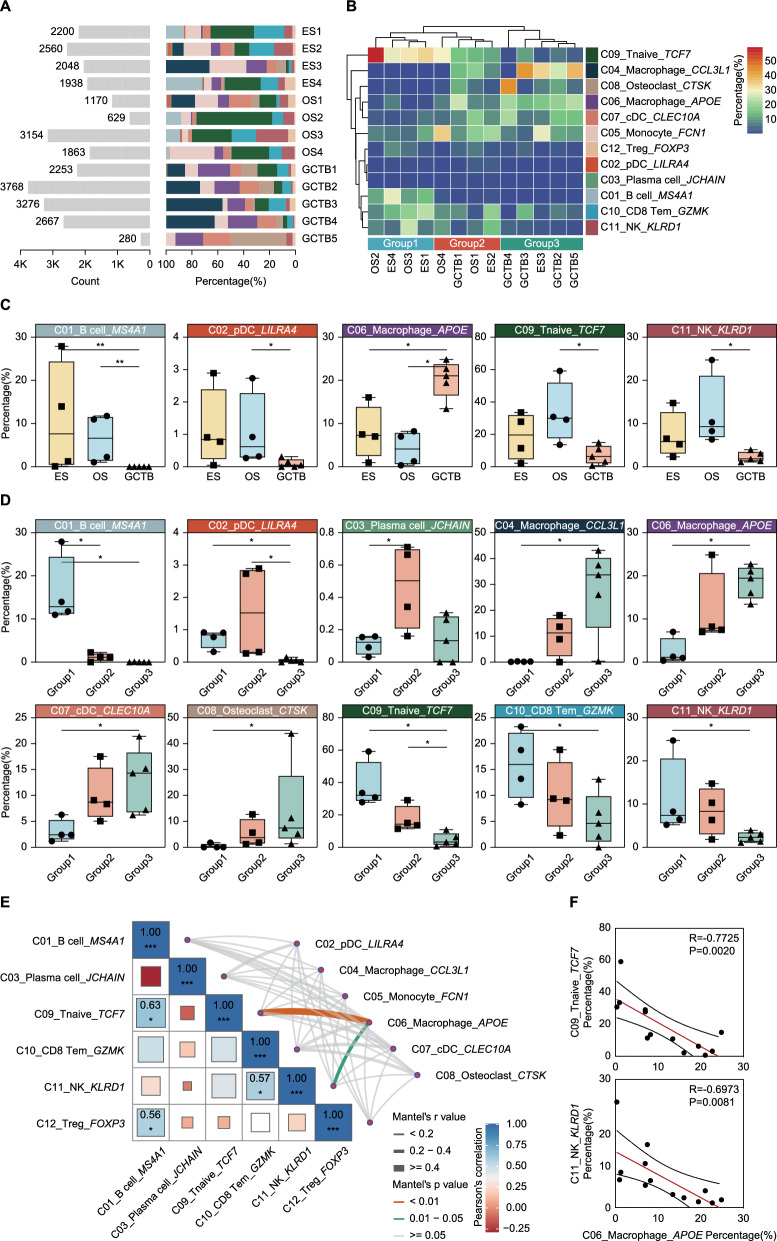
Immune cell composition and functional correlation in bone tumor samples. **A** Bar plot showing the distribution of immune cell clusters across individual tumor samples. **B** Cluster dendrogram displaying the similarity between tumor samples. Based on immune cell composition, samples are categorized into three groups. **C** Boxplots grouped by tumor type (OS, ES, and GCTB) showing the distribution of immune cell clusters with significant differences. **D** Boxplots grouped by the immune classification identified in **B**. **E** Matrix plot showing Mantel’s test results for pairwise correlation between immune cell clusters based on gene expression profiles. The correlation strength (r value) and statistical significance (P-value) are represented by color intensity. **F** Scatter plots showing the correlation of C06_Macrophage_*APOE* with C09_Tnaive_*TCF7* and C11_NK_*KLRD1*. The X-axis represents the percentage of C06_Macrophage_*APOE*, while the Y-axis shows the percentage of the respective clusters

Clustering analysis of immune subpopulations identified three immunological subtypes (Group 1–3) across bone tumor samples, reflecting the heterogeneity of their immune microenvironments (Fig. 2A, B). Statistical analysis revealed significant differences in immune subpopulation distributions among tumor types (Fig. 2C). B cells (C01) were significantly enriched in ES and OS compared to GCTB (P < 0.01), while pDCs (C02), naïve T cells (C09), and NK cells (C11) were most abundant in OS (P < 0.05), highlighting active adaptive and innate immune responses in this subtype. Conversely, C06 tissue-remodeling macrophages, predominantly enriched in GCTB, were associated with metabolic regulation and extracellular matrix remodeling (P < 0.05). This observation aligns with prior studies suggesting a critical role for myeloid cells in regulating the tumor matrix and bone remodeling in GCTB.

Analysis of immune subpopulation distributions within groups further defined the characteristics of each subtype (Fig. 2D). Group 1 (comprising two ES and two OS samples) represented a lymphoid-dominated immune phenotype, with significant enrichment of B cells (C01) and higher levels of pDCs (C02), naïve T cells (C09), memory T cells (C10), and NK cells (C11) compared to other groups. This phenotype suggests a microenvironment dominated by adaptive immune activation, consistent with prior findings linking enhanced lymphoid infiltration to anti-tumor immunity in osteosarcoma. Group 2, composed of two OS samples, one ES sample, and one GCTB sample, exhibited an intermediate level of immune infiltration. Although pDCs (C02) and naïve T cells (C09) were less enriched compared to Group 1, their levels were remained higher than those in Group 3. Additionally, Group 2 displayed significant enrichment of plasma cells (C03), indicative of active humoral immune responses. These findings align with previous research highlighting the role of plasma cells in antibody-mediated tumor immunity. Group 3, predominantly composed of GCTB samples (4 cases) and one ES sample, exhibited a myeloid-dominated phenotype characterized by significant enrichment of macrophages (C04 and C06), classical dendritic cells (C07), and osteoclasts (C08). This subtype reflects a tumor microenvironment characterized by inflammation, tissue remodeling, and immune suppression, consistent with prior findings on the critical roles of myeloid cells in GCTB-associated bone resorption and immunosuppression.

Correlation analysis revealed cooperative interactions within lymphoid cells (Fig. 2E). B cells (C01) exhibited a strong positive correlation with naïve T cells (C09) (R = 0.63, P < 0.05) and regulatory T cells (C12) (R = 0.56, P < 0.05), suggesting that B cells may support adaptive immunity through collaboration with naïve T cells while maintaining immune homeostasis via interactions with regulatory T cells. Additionally, memory T cells (C10) and NK cells (C11) displayed a significant positive correlation (R = 0.57, P < 0.05), indicative of functional cooperation in enhancing cytotoxic anti-tumor responses. These findings are consistent with previous research demonstrating that memory T cells enhancing NK cell activity to promote tumor cell killing. In contrast, C06 macrophages exhibited negative correlations with naïve T cells (C09) (R = −0.7725, P = 0.0020) and NK cells (C11) (R = −0.6973, P = 0.0081), reinforcing their immunosuppressive role (Fig. 2F). This suppression may be mediated through inhibitory signaling pathways or extracellular matrix remodeling, which hinders lymphoid cell infiltration and activation.

Taken together, we underscore the distinct immune landscapes of bone tumors and reveal dynamic interactions between immune subtypes. While cooperative interactions among lymphoid cells enhance adaptive and cytotoxic immunity, myeloid-driven immunosuppressive mechanisms counteract lymphoid-mediated immune activation.

### Metabolic and functional plasticity of T and NK cells in the bone tumor microenvironment

**Fig. 3:**
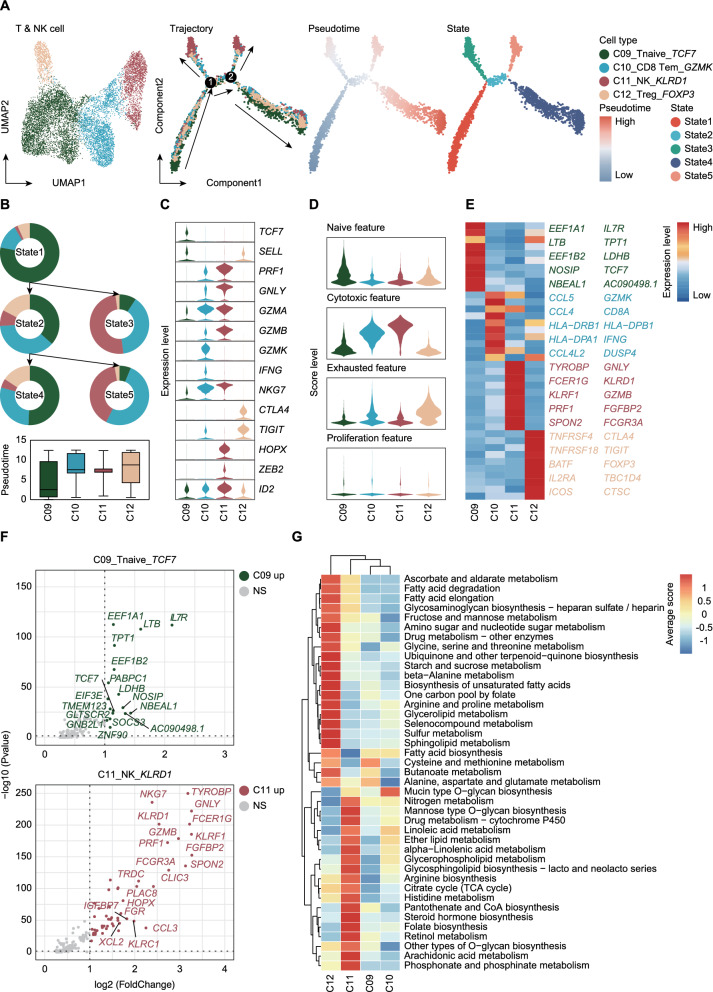
Trajectory analysis and functional features of T and NK cells in bone tumors. **A** UMAP plot and pseudotime trajectory of T and NK cells. The trajectory is colored by pseudotime (from low to high) and state transitions (State 1 to State 5). **B** The upper panel shows the developmental tree of T and NK cells, with each node represented as a pie chart displaying the composition of cell types from different states along the trajectory. The lower panel consists of boxplots depicting the pseudotime distribution of each cluster. **C** Violin plots showing the expression of functional genes in T and NK cells. **D** Violin plots displaying naïve, cytotoxic, exhausted, and proliferation scores for each cluster. **E** Heatmap presenting the top 10 DEGs for each T and NK cell cluster. **F** Volcano plot identifying upregulated genes in C09_Tnaive_*TCF7* and C11_NK_*KLRD1*. Genes with significant fold changes and adjusted P-values are highlighted. **G** Clustering heatmap showing the enriched metabolic pathways for each cluster

To elucidate the immune landscape and cellular dynamics of T and NK cells within the bone tumor microenvironment, we employed pseudotime analysis to map their differentiation trajectories across distinct states (Fig. 3A). Five differentiation states (State 1–5) were identified, representing transitions from naïve to effector and regulatory phenotypes. State 1, primarily composed of naïve T cells (C09), exhibited high expression of *TCF7* and *SELL*, highlighting their roles in early differentiation and antigen recognition. State 2, a transitional phase, demonstrated an increasing presence of memory T cells (C10), characterized by *GZMK*, *CCL5*, and *NKG7* expression, indicative of enhanced cytotoxic and effector functions, Despite its mixed lymphoid composition, this state suggested a shift toward an activated immune phenotype. State 3 and State 5 were primarily composed of NK cells (C11), reflecting a distinct differentiation trajectory due to the phylogenetic divergence between NK cells and T cells. These states were defined by high expression of *TYROBP*, *GNLY*, and *PRF1*, underscoring the innate cytotoxic capabilities of NK cells. State 4, dominated by regulatory T cells (C12), emerged as the most prominent population along the differentiation continuum, marked by high *FOXP3*, *CTLA4*, and *IL2RA* expression. This state encapsulated the dynamic equilibrium between immune exhaustion and proliferation, functioning as a regulatory hub within lymphoid differentiation (Fig. 3B, C).

Gene set enrichment analysis revealed metabolic specializations associated with these functional states (Fig. 3D, F). Naïve T cells (C09) were enriched in pathways such as cysteine and methionine metabolism and butanoate metabolism, indicating a reliance on amino acid and sulfur metabolism to sustain cellular readiness and oxidative balance (Fig. 3G) [14]. In contrast, NK cells (C11) exhibited significant enrichment in glycerophospholipid metabolism, linoleic acid metabolism, and the citrate cycle (TCA cycle), with high expression of *PRF1*, *KLRF1*, and *SPON2*, essential for cytotoxic degranulation. Additionally, their enrichment in arginine biosynthesis and ether lipid metabolism highlighted their metabolic adaptability to nutrient fluctuations in the tumor microenvironment (Fig. 3F, G). This metabolic diversity underscores the functional disparities between quiescent T cells and highly active NK cells.

In summary, T and NK cells within the bone tumor microenvironment exhibit dynamic functional and metabolic plasticity, balancing immune activation and suppression.

### Dynamic differentiation and functional specialization of myeloid cells in the bone tumor microenvironment

**Fig. 4:**
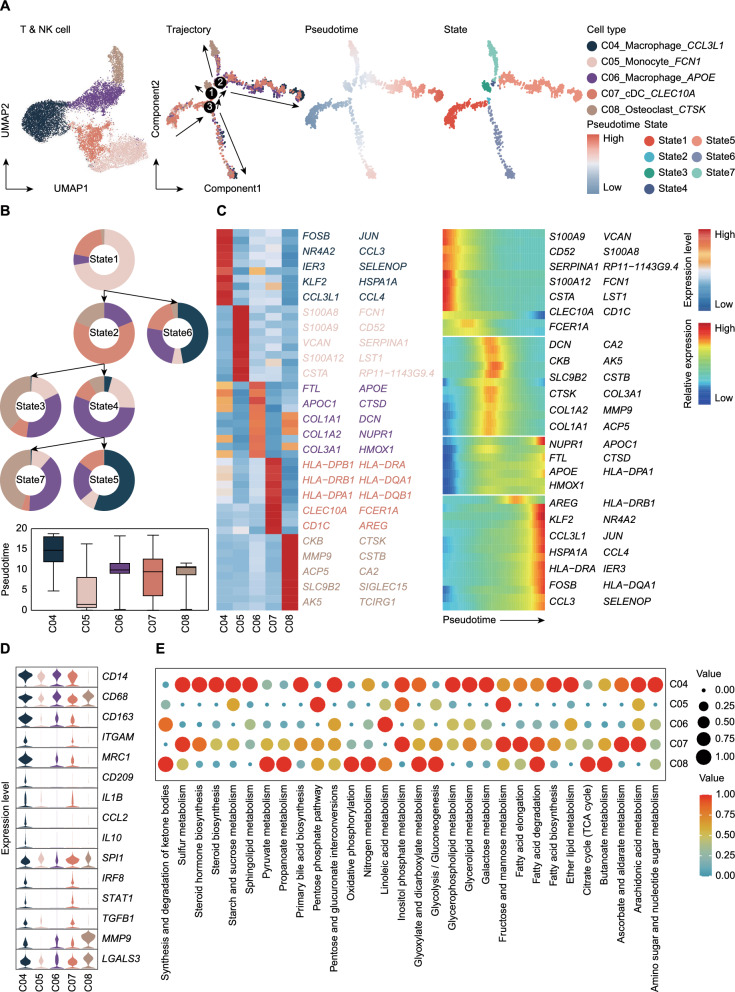
Pseudotime trajectory and metabolic specialization of myeloid cells. **A** UMAP and trajectory plot illustrating the pseudotime of myeloid cells. **B** Developmental tree and pseudotime boxplots of myeloid cells. **C** Heatmaps showing the DEGs for myeloid cell clusters. The left panel highlights the DEGs for each cluster, while the right panel visualizes the dynamic expression of these DEGs along the pseudotime trajectory. **D** Violin plots illustrating the expression levels of functional genes across myeloid cell clusters. **E** Dot plot depicting the enriched metabolic pathways for each myeloid cell cluster

To dissect the differentiation dynamics of myeloid cells in the bone tumor microenvironment, we performed pseudotime analysis and identified seven distinct differentiation states (State 1–7), spanning from early immune responses of monocytes to the tissue-remodeling functions of osteoclasts (Fig. 4A). The diverse differentiation trajectories of myeloid cells were closely linked to their functional and metabolic specializations, highlighting their critical roles in shaping the tumor microenvironment.

State 1 was predominantly composed of monocytes (C05) that highly expressed *FCN1* and *S100A* family genes (Fig. 4B, C). These genes are associated with inflammatory signaling and early immune activation, emphasizing the pivotal role of monocytes in initiating immune responses [15]. By contrast, State 2 was enriched in classical dendritic cells (C07), characterized by high expression of *CLEC10A*, *HLA-DRA*, and *CD1C* (Fig. 4B, C), reflecting their essential roles in antigen presentation and adaptive immune activation [16]. As differentiation progressed, State 3 and 7 were dominated by osteoclasts (C08), which highly expressed *CTSK*, *MMP9*, and *ACP5* (Fig. 4B, C). These genes highlight the osteoclasts’ key functions in extracellular matrix degradation and bone remodeling [17]. Metabolic analysis revealed significant enrichment in the citrate cycle and oxidative phosphorylation pathways (Fig. 4E), suggesting that osteoclasts rely on energy-intensive metabolic processes to sustain bone resorption. State 4 was primarily composed of macrophages (C06) that predominantly expressed *APOE* and *HMOX1* (Fig. 4B, C), reflecting their involvement in lipid metabolism and anti-inflammatory functions [10]. Metabolic analysis indicated significant enrichment in synthesis and degradation of ketone bodies and linoleic acid metabolism (Fig. 4E), suggesting that C06 macrophages regulate immune suppression and tissue repair through lipid metabolic reprogramming [18]. State 5 and State 6 were enriched in another macrophage subset (C04), which highly expressed genes associated with complement activation, pro-inflammatory signaling, and chemotaxis (Fig. 4D). In contrast, C06 macrophages exhibited minimal expression of these genes, reinforcing their functional divergence. This discrepancy underscores the pro-inflammatory and highly activated nature of C04 macrophages, while C06 macrophages are more specialized in tissue repair and metabolic regulation.

Myeloid cells in the bone tumor microenvironment exhibit significant functional heterogeneity and dynamic differentiation. From the immune activation roles of monocytes to the matrix degradation functions of osteoclasts, and the divergent roles of macrophages in inflammation and repair.

### Cellular interactions and metabolic-signaling regulation in the bone tumor microenvironment

In the bone tumor microenvironment, myeloid and lymphoid cells orchestrate tumor immune regulation through complex intercellular interactions and metabolic remodeling. Building upon previous findings, we conducted a comprehensive analysis of intercellular signaling networks, integrating metabolic pathway characteristics to reveal key communication mechanisms among monocytes, macrophages, osteoclasts, T cells, and NK cells, with a particular focus on the FN1 signaling pathway.

**Fig. 5:**
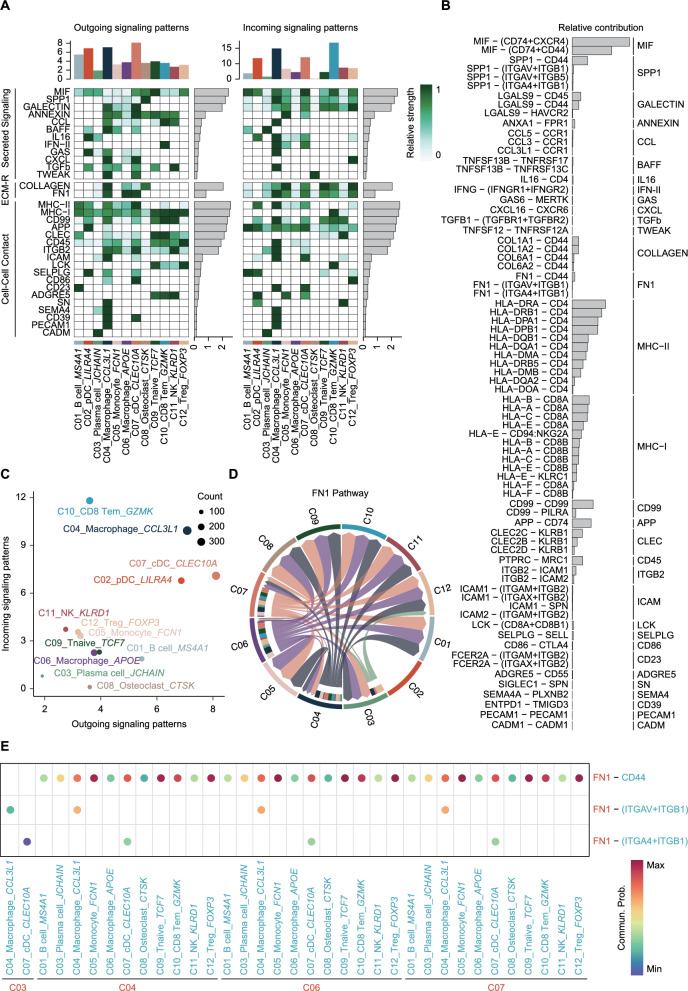
Intercellular communication and signaling networks in bone tumor microenvironment. **A** Heatmap of outgoing and incoming signaling patterns for each immune cell cluster. **B** Bar plots summarizing the overall communication strength of major pathways across immune cell clusters. **C** Scatter plot showing the relative contribution of different immune cell clusters to shaping the immune microenvironment (outgoing signaling strength) and receiving environmental signals (incoming signaling strength). **D** Circle plots displaying the detailed intercellular communication network for FN1 signaling pathways. **E** Dot plot illustrating the specific interaction mechanisms within the FN1 signaling pathway

Signal transmission analysis indicated that the immune regulation and tissue remodeling functions of myeloid cells are closely associated with their metabolic state (Figs. 5A, B). Osteoclasts (C08) secreted SPP1 interacting with integrin receptors (ITGAV + ITGB1, ITGAM + ITGB2), thereby forming robust signaling connections. These interactions not only facilitated the migration and activation of lymphoid cells but also enhanced osteoclast integration into the local microenvironment during bone matrix degradation [19]. Among macrophage subsets (C04 and C06), distinct functional roles were observed: C04 macrophages mediated pro-inflammatory signals via CCL3L1-CCR1 interactions, while C06 macrophages regulated immune suppression through APOE and lipid metabolism pathways, reflecting their anti-inflammatory and tissue-repairing functions.

Lymphoid cells, including T cells (C09, C10) and NK cells (C11), also played critical roles in signal transmission and metabolic remodeling (Fig. 5A, C). T cells, through high IFNG expression, formed a feedback loop with myeloid cells, driving immune activation [20] and further enhancing intercellular adhesion and migration via FN1 signaling. The FN1 signaling pathway played a central regulatory role in the interaction between myeloid and lymphoid cells (Figs. 5D, E). FN1 engaged integrin receptors such as ITGA4 + ITGB1, ITGAV + ITGB1, and CD44 to modulate cell adhesion, migration, and signal transmission [21, 22]. This pathway established significant bidirectional signaling axes between macrophages (C04 and C06) and T cells (C09 and C12), providing structural and functional support for the coordinated activity of various cell types within the microenvironment. Furthermore, FN1 signaling was significantly supporting cellular energy demands and maintaining immune suppression and homeostasis within the tumor microenvironment by regulating immune cell function [23, 24].

In conclusion, we integrated multidimensional analyses of intercellular signaling and metabolic remodeling, revealing the dynamic communication mechanisms between myeloid and lymphoid cells in the bone tumor microenvironment. As a key hub, the FN1 signaling pathway not only bridges physical and functional intercellular interactions but also drives metabolic adaptation to maintain tumor microenvironment homeostasis and immune dysfunction.

## Discussion

The development of bioinformatics algorithms has significantly contributed to advancing life science research [25–27]. With the rapid evolution of sequencing technologies, single-cell RNA sequencing (scRNA-seq) has become an indispensable tool for dissecting the immune microenvironment of tumors [28, 29]. Computational methods enable the efficient processing and visualization of high-dimensional data [30]. Through scRNA-seq analysis, researchers have gained a comprehensive understanding of the distribution and subtype-specific characteristics of immune cells across various cancer types [31–33]. However, single-cell sequencing also has inherent limitations, including limited coverage depth and the inability to capture certain rare cell subpopulations. Nevertheless, it remains a powerful approach for studying cellular heterogeneity and immune dynamics in tumors.

In recent years, several single-cell studies have provided valuable insights into bone-related tumors. For example, Shenglin Wang et al. constructed a high-resolution cellular atlas of the bone metastatic microenvironment in NSCLC, revealing the central role of cellular senescence in bone metastasis [34]. Their study identified the SOX18 and SPP1 signaling pathways as potential therapeutic targets and highlighted the role of CD4Tstr cells in immunosuppression and angiogenesis. Similarly, targeting cancer-associated fibroblasts (CAFs) has emerged as a key research focus in bone tumors. Xin Huang et al. applied single-cell sequencing to analyze the tumor microenvironment of recurrent osteosarcoma, identifying SERPINE1-expressing CAFs as a critical driver of tumor recurrence by promoting CAF activation, enhancing the epithelial-to-mesenchymal transition (EMT) process, and modulating macrophage polarization [35]. Furthermore, their research characterized a distinct LOX-high-expressing CAF phenotype, demonstrating its essential role in osteosarcoma progression [36].

The tumor immune microenvironment (TME) plays a crucial role in cancer progression, clinical classification, and precision therapy. Previous studies have outlined four key strategies for enhancing T-cell anti-tumor activity by modulating immune checkpoints through nanotechnology-based delivery systems to counteract T-cell exhaustion [37]. These systems can also be leveraged to reprogram macrophages, enhancing the stability of METTL14 in vivo, inhibiting the TLR4 pathway, regulating macrophage polarization, and remodeling the TME to improve anti-tumor responses [38]. In contrast, our study focused on the metabolic regulation of immune cells within the TME. Using scRNA-seq, we systematically characterized the immune landscape of bone tumors, including Ewing’s sarcoma (ES), osteosarcoma (OS), and giant cell tumor of bone (GCTB). Our findings revealed extensive immune heterogeneity and functional specialization, highlighting distinct immune dynamics orchestrated by lymphoid and myeloid populations.

Notably, we identified macrophage subsets with distinct functional properties, such as the pro-inflammatory C04 and the tissue-remodeling C06 macrophages, underscoring their dual roles in promoting inflammation and immunosuppression, which are intimately linked to tumor progression and extracellular matrix remodeling. Subtype-specific immune profiles were also observed, with ES and OS tumors exhibited lymphoid-dominant microenvironments enriched in B cells, naïve T cells, and NK cells, indicative of active adaptive and innate immune responses. Conversely, GCTB displayed a myeloid-dominated phenotype, characterized by significant enrichment of osteoclasts and macrophages, emphasizing the role of myeloid cells in tissue remodeling and immunosuppression. These findings refine our understanding of bone tumor subtypes and suggest the necessity of tailored immunotherapeutic strategies that target the unique immune compositions of each tumor type.

Metabolic analysis further revealed that immune cells within the bone tumor microenvironment adapt their metabolic pathways to meet distinct functional demands. Naïve T cells relied on amino acid metabolism to sustain activation potential [14], while NK cells exhibited preferential enrichment in lipid metabolism and the TCA cycle to support cytotoxic activity. Additionally, macrophage subsets displayed divergent metabolic profiles, linking lipid metabolism to their roles in tissue repair and immune suppression [10]. These findings emphasize the intricate interplay between cellular function and metabolic plasticity, providing a rationale for targeting immune cell metabolism as a strategy to modulate the TME.

The TME is a highly dynamic and complex system that plays a pivotal role in cancer progression, immune regulation, and therapeutic resistance [39, 40]. Among the key regulators of the TME, fibronectin 1 (FN1), a major extracellular matrix (ECM) protein, has emerged as a critical modulator of tumor-immune interactions. A study on prostate cancer bone metastasis identified FN1 as a key player in macrophage-induced anti-androgen resistance, proposing the FN1-integrin-SRC signaling axis as a potential therapeutic target for metastatic castration-resistant prostate cancer [41]. Furthermore, studies on head and neck tumors have demonstrated that high FN1 expression is significantly associated with poor prognosis and higher pathological grade in HNSCC patients. The autophagy-lysosome degradation pathway of FN1 has been implicated in regulating EMT in HNSCC [42]. Consistently, our study identified FN1 signaling as a central pathway modulating immune cell dynamics, with key interactions involving integrin receptors such as ITGA4 + ITGB1, ITGAV + ITGB1, and CD44. Similarly, studies in breast and kidney cancers have also validated these findings [24, 43].

In conclusion, by delineating subtype-specific immune dynamics and identifying critical cellular interactions, such as the FN1-mediated signaling network, our study highlights novel opportunities for immunotherapy. Targeting specific metabolic pathways in immune cells may enhance their anti-tumor activity or attenuate immunosuppressive mechanisms within the TME. Moreover, integrating metabolic profiling into diagnostic protocols could facilitate the early identification of patients most likely to benefit from personalized immunotherapy. Nevertheless, our study has certain limitations, including a relatively small sample size, necessitating further validation in larger patient cohorts. Additionally, as the data were derived from multiple datasets, potential discrepancies in treatment backgrounds and patient heterogeneity must be considered. Future research will focus on elucidating the causal relationships between metabolic pathways and immune cell function, conducting clinical trials to facilitate translational applications, and evaluating the real-world efficacy of metabolic interventions.

## Supplementary Information

Supplementary Material 1

Supplementary Material 2

Supplementary Material 3

Supplementary Material 4

Supplementary Material 5

Supplementary Material 6

Supplementary Material 7

Supplementary Material 8

Supplementary Material 9

Supplementary Material 10

Supplementary Material 11

Supplementary Material 12

## Data Availability

The datasets generated and analyzed in this study can be found in online repositories. The accession number can be found in the article/Supplementary Material. Further inquiries can be directed to the corresponding authors.
